# Optimization of a CUPRAC-Based HPLC Postcolumn Assay and Its Applications for *Ginkgo biloba* L. Extracts

**DOI:** 10.1155/2015/280167

**Published:** 2015-07-05

**Authors:** Laura Rimkiene, Liudas Ivanauskas, Asta Kubiliene, Konradas Vitkevicius, Guoda Kiliuviene, Valdas Jakstas

**Affiliations:** ^1^Department of Analytical and Toxicological Chemistry, Faculty of Pharmacy, Medical Academy of Lithuanian University of Health Sciences, Eiveniu Street 4, LT-50161 Kaunas, Lithuania; ^2^Department of Pharmacognosy, Faculty of Pharmacy, Medical Academy of Lithuanian University of Health Sciences, Eiveniu Street 4, LT-50161 Kaunas, Lithuania

## Abstract

The aim of the present work was to improve and validate the HPLC-CUPRAC postcolumn method for the evaluation of active antioxidant markers from the acetonic extracts of *Ginkgo biloba* leaves. Improvement of the HPLC online assay was performed by evaluating the suitable loop temperature, the reaction loop length, and the impact of flow rate. Separation of the analytes was performed by the HPLC method on an ACE C18 analytical column using a gradient elution program. The separated antioxidant markers in the extracts reacted with copper(II)-neocuproine (Cu(II)-Nc) reagent in the postcolumn reaction coil. The reagent was reduced by antioxidants to the copper(I)-neocuproine (Cu(I)-Nc) chelate with a maximum absorption at 450 nm. Validation experiments confirmed sufficient precision, sensitivity, and effectiveness of the corresponding method, which could be used for further evaluations of active antioxidant compounds in similar plant materials.

## 1. Introduction

The pathogenesis of most illnesses such as cancer, cardiovascular diseases, Alzheimer's disease, diabetes, and nervous and immune system diseases includes oxidative stress, an imbalance of oxidizing (usually active oxygen compounds) and reducing compounds [1, 2]. Antioxidants can be prescribed in the case of the above conditions [3]. The curative effect and quality indices of pharmaceutical products should be completely reciprocal. However, the analytical methods that would provide a quantitative and bioequivalent assessment of the distribution of antioxidant markers in pharmaceutical and composite plant products are lacking. The application of modern marker oriented analysis methods is indispensable in equivalence tests and for the quality control assurance of phytopreparations.

Currently, only online methods that combine HPLC distribution and postcolumn reaction are promising implements for pharmaceutical-scale characterization of antioxidant active markers [4]. The validated HPLC postcolumn methods enable an enriched evaluation with complex “fingerprint” chromatograms that indicate not only the phytochemical composition of medicinal plant raw material or its preparation but also the distribution of the compounds with antioxidant activity in the preparation. These systems allow control of antioxidant stability and the establishment of optimal storage conditions for the raw material or preparation [5–7]. The selection of conditions for postcolumn reaction parameters is an important step in the development and optimization of these methodologies as it has a major impact on the accuracy and reliability of the final results.

Leaves of* Ginkgo biloba* have long been used for medical purposes. The extracts from* Ginkgo biloba* leaves have become a very popular plant medicine and herbal supplement due to their antioxidant potential, with the potential to treat Alzheimer's disease and dementia [8, 9]. According to the literature, quercetin, kaempferol, and isorhamnetin are assigned to be fingerprints which can be used to evaluate the quality and authenticity of* Ginkgo biloba* extracts and dosage forms. Therefore, aglycones described above which are used in “fingerprint" analysis were chosen for our further investigations.

Our work aims to identify the essential conditions of the HPLC-CUPRAC postcolumn method for the assessment of potential reducing compounds that exist in the extracts of* Ginkgo biloba* leaves. The CUPRAC reagent has been chosen for its advantages against other reagents used for the establishment of antioxidant activity on the basis of the electron transfer reaction. The TEAC values obtained with CUPRAC postcolumn assay are similar to the findings of the ABTS/TEAC reference method but these two antioxidant activity assessment methods are based on different mechanisms of action. CUPRAC method seems to be the more robust, because reagent is more selective, CUPRAC absorbance calibration curve is linear over a wide concentration range, and postcolumn chemical reaction is generally faster unlike ABTS method [10]. For these reasons, CUPRAC reagent has analytical advantages in analysing plant material of unknown composition.

## 2. Materials and Methods 

### 2.1. Plant Material

*Ginkgo biloba* phenotypes from the seven different collections were studied. The leaf samples were collected from Siauliai (sample 1) and Klaipeda (sample 2) botanical gardens, Kaunas (sample 3) botanical garden of Vytautas Magnus University, and Lazdijai (sample 4), Kazlu Ruda (sample 5), Silute (sample 6), and Utena (sample 7) districts of Lithuania in September 2013 (voucher specimens: 2002/0925, 2005/0926, 1936/0503, 2008/0625, 2003/1005, 1915/0510, and 2004/0813, resp.).

### 2.2. Preparation of Extracts

Samples of* Ginkgo biloba* leaves were prepared according to the method described in the European Pharmacopoeia monograph (Ph. Eur. 01/2011:1828) [11] of* Ginkgo* leaf (Ginkgonis folium). The extracts were produced by weighing 2.5 g of material (precise weight) and adding the solvent up to a total volume of 50.0 mL. The samples of raw material were extracted in a 60% acetone/water mixture (v/v) by heating under a reflux condenser twice for 30 min. 50.0 mL of prepared solution was evaporated till the elimination of acetone. Remaining extract was transferred to 50.0 mL vial, rinsed with 30.0 mL of methanol and 4.4 mL of hydrochloric acid, and diluted with water to the 50.0 mL mark. 10.0 mL of the supernatant liquid was placed in a 10.0 mL brown-glass vial, which was closed with a rubber seal and aluminium cap and heated on the water bath for 25 min. The extracts were filtered through 0.22 *μ*m nylon syringe filters (Carl Roth GmbH & Co. KG, Germany).

### 2.3. HPLC-PDA Conditions

The analysis was conducted with a Waters 2695 chromatographer (Waters Corporation, Milford, USA) and a Waters 996 photodiode matrix detector (Waters Corporation, Milford, USA). Active compounds were separated with a 150 × 4.6 mm, 5 *μ*m ACE C18 column (Advanced Chromatography Technologies, Scotland) which was kept in the external thermostat at a constant temperature of 25°C. The injection volume was 10 *μ*L, and the elution flow rate was 1.0 mL min^−1^. Eluents A and B were used for the gradient elution. Solution A was water containing 0.3 g L^−1^ orthophosphoric acid (pH 2.0) and solution B was methanol. The following gradient was used: 0 min to 1 min with 60% A and 40% B; 1 min to 20 min from 60% to 45% A and from 40% to 55% B; 20 min to 21 min from 45% to 0% A and from 55% to 100% B; 21 min to 25 min with an isocratic elution of 100% B; 25 min to 30 min from 0% to 60% A and from 100% to 40% B. Chromatographic peaks were identified according to the concurrence of analytes and standard compound retention times and UV absorption spectrum within 210–400 nm. The separated active compounds were analysed at a 370 nm wavelength to ensure their maximum absorption.

### 2.4. Online HPLC-CUPRAC Assay with Postcolumn Detection

After applying the HPLC-PDA detection system, the mobile phase containing the analytes was fed into a reaction coil through a mixing tee where the CUPRAC reagent was supplied at the same time by a Gilson pump 305 (Middleton, USA). Reaction coils made of TFE (Teflon) of 3, 5, and 10 m length, 0.25 mm i.d. and 1.58 mm o.d., were used. The system with CUPRAC solution was monitored as follows: temperature range at 25–55°C and 0.2, 0.3, 0.4, and 0.5 mL min^−1^ flow rates of the reagent were set.

In this study, the CUPRAC solution was prepared following the method described by Çelik et al. [12]. After the reaction with the antioxidant, CUPRAC reagent was reduced to the yellow colour complex Cu(I)-Nc. The increase of absorption was fixed with a Waters 2487 UV/VIS detector (Waters Corporation, Milford, USA) and positive peaks of antioxidant impact by individual compounds at the 450 nm wavelength were recorded in the chromatogram.

### 2.5. Antioxidant Activity Assessment

The antioxidant activity of sample compounds was assessed by standard antioxidant Trolox. Calibration curves of Trolox (concentrations range 0.59–605 *μ*g mL^−1^) were made in ABTS postcolumn assay. The copper reducing capacity of antioxidant active compounds in* Ginkgo biloba* extracts was expressed as Trolox equivalent antioxidant capacity (TEAC). TEAC corresponds to Trolox amount (*μ*mol), which, under the same experimental conditions, possesses identical antioxidant activity as 1 g of plant material. TEAC was calculated by the following formula:

(1) $$\begin{array}{c} \text{TEAC}=\frac{S_{\text{comp.}}-b}{a}\;\left(\;\mu M\;\right)\times \frac{V_{\text{sampl.}}\;\left(L\right)}{m_{sampl.}\;\left(g\right)},\;\;\left(\;\mu mol/g\;\right) \end{array}$$

*S*_comp._ is the peak area of antioxidant active compound in the postcolumn chromatogram; *a* is slope, and *b* is intercept from Trolox calibration curve regression equation; *V*_sampl._ is the volume of herbal raw material extract; *m*_sampl._ is the weighed (precise) quantity of herbal raw material.

### 2.6. Reagents and Chemicals

The methanol of HPLC grade was supplied by Roth GmbH (Karlsruhe, Germany), and the ethanol was produced by Stumbras (Kaunas, Lithuania). The acetone (HPLC grade), hydrochloric acid (37%), 2,9-dimethyl-1,10-phenanthroline (neocuproine, 98%), copper(II) chloride dihydrate (99%), and orthophosphoric acid (85%) were purchased from Sigma-Aldrich GmbH (Buchs, Switzerland). Trolox (98%) was obtained from Fluka Chemie (Buchs, Switzerland). The reference substances, kaempferol (99%) and isorhamnetin (99%), were purchased from Extrasynthese (Genay, France) and quercetin (99.49%) was purchased from HWI ANALYTIK GmbH (Rülzheim, Germany). Ultrapure water was purified with a Millipore water cleaning system (Bedford, USA).

### 2.7. Data Analysis

The statistical package SPSS 17 (SPSS Inc., Chicago, USA) was used for statistical processing and evaluation of the data. One-way analysis of variance (ANOVA) was used to estimate significant differences among the variables using different reaction coil lengths and TEAC values. MS Excel (Microsoft, USA) was used for graphical presentation of the results.

## 3. Results and Discussion

### 3.1. Technical Aspects of the HPLC-CUPRAC Postcolumn Assay

**Figure 1:**
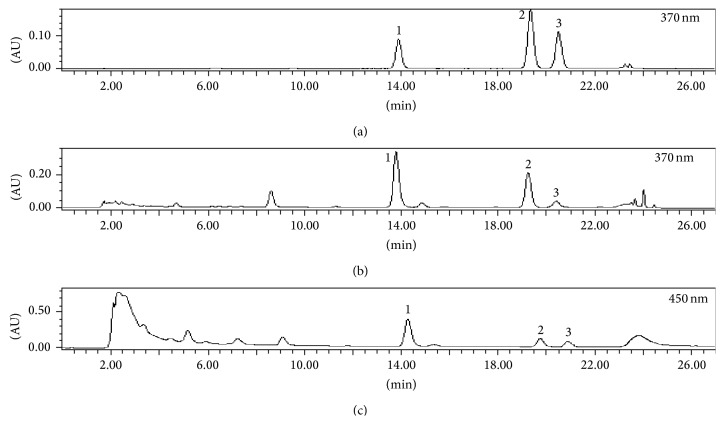
Combined chromatograms of chromatographic elution of a mixture of flavonoid standards (a),* Ginkgo biloba* L. leaf extract (b), and postcolumn reaction with CUPRAC with* Ginkgo biloba* L. leaf extract (c). For exact compound refer to Table 2.

In the course of experimental investigations, one scientific group has applied the HPLC-CUPRAC postcolumn method for the identification of antioxidant activity compounds in* Camellia sinensis*,* Mentha*,* Origanum majorana*, and* Sambucus nigra* extracts [12, 13]. The literature presents descriptions of HPLC-CUPRAC postcolumn methods that are based on colour formation after the reaction with antioxidants, where the increase in absorption is monitored as negative peaks in the postcolumn chromatogram. In the course of our investigation, the postcolumn detection was initially performed with a Waters 996 photodiode matrix detector (Waters, Milford, MA, USA) in the sphere of 210–600 nm. The maximum absorption of the CUPRAC reagent mixture after the reaction with analytes of reducing characteristics (quercetin, kaempferol, and isorhamnetin) was established at the 450 nm wavelength. After obtaining the investigation data which confirmed the research results [12–14], our further investigation involved postcolumn detection at the 450 nm wavelength. However, the change of absorption in the postcolumn chromatogram was monitored as a positive peak (Figure 1), which contradicts the works of Çelik et al. [12, 13].

The structure and concentration of the reagents, probe, postcolumn flow rate, tubing length or reaction time, and system temperature are considered to be the main factors in postcolumn detection [15, 16]. Potential narrowing of the loop diameter is an actual aspect of HPLC postcolumn methods. Dapkevicius et al. [17] and Çelik et al. [12] stated that, during the derivatization of the postcolumn reaction with DPPH reagent, narrowing of the reaction loop diameter is possible due to the deviation by mixtures of postcolumn system solutions from neutral conditions and an excessive concentration of antiradical reagents or analytically impure reagents. Our investigation showed that the formation of sediments during the gradient analysis gave rise to clogging of the reaction coil. In this way, the undesirably high pressure and instability at baseline are caused in the HPLC-CUPRAC postcolumn system. This in turn adversely affected the detection of the reaction mixture absorption. Microscopic analysis of the sediment showed formation of blue colour crystals that are soluble in strong mineral acids and diluted acid solutions but insoluble in organic solvents.

Therefore, the derivatizing reagent's flow rate, reaction loop, and temperature should be considered in the selection of characteristics that would prevent formation of the sediments that impede the detection of the reaction mixture absorption.

### 3.2. Assessment of CUPRAC Postcolumn Assay Characteristics

Certain temperatures are used in the postcolumn reaction, but there is no improvement in the methodological justification for the chosen temperature limit for antioxidant activity assays on plant materials [13, 18]. The investigation conducted at the temperature higher than 25°C showed that increasing the temperature of the postcolumn reaction also increases backward pressure in the postcolumn system. Despite the chosen flow rate and reaction loop length of the CUPRAC reagent, the formation of blue colour crystals was observed at increasing temperatures in the reaction loop. Therefore, the chosen reactor temperature for further experimental investigations is 25°C. It was experimentally confirmed that this temperature assures stability of the postcolumn system for a 72-hour series analysis course. For practical work, application of 50°C is not recommended in a CUPRAC postcolumn system, whereas temperatures no higher than 25°C are suggested when taking the analytical objectives into account.

Having evaluated the ratio between peak height and baseline (*S*/*N*), Çelik et al. [12] established 0.5 mL min^−1^ to be the optimal flow rate of CUPRAC solution to the postcolumn system. However, our investigation resulted in different experimental data. When the flow of eluents is 1 mL min^−1^, the rate of the CUPRAC solution supply to postcolumn system exceeding 0.3 mL min^−1^ is not suitable for routine experimental work.

**Figure 2:**
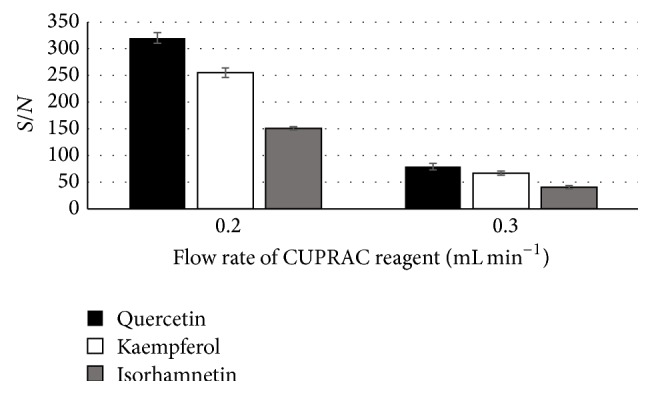
Dependence of peak height-to-baseline noise ratio (*S*/*N*) on CUPRAC solution flow rate. The following standards were used: quercetin, 0.074 mg mL^−1^; kaempferol, 0.084 mg mL^−1^; isorhamnetin, 0.085 mg mL^−1^ (injected volume: 10 *μ*L; *λ* = 370 nm).

In the course of the investigation, the rate of CUPRAC solution supply to the postcolumn system at 0.2 mL min^−1^ was established to be the best for the planned experimental investigations (Figure 2). At this flow rate, the postcolumn system is stable, the CUPRAC reagent has sufficient capacity to control antioxidant compounds of various activity, and the achieved *S*/*N* values of the standard compounds are significantly better than those at the reagent supply at 0.3 mL min^−1^. 0.2 mL min^−1^ was the selected value for this factor due to higher flow rates causing overpressure in the system.

**Figure 3:**
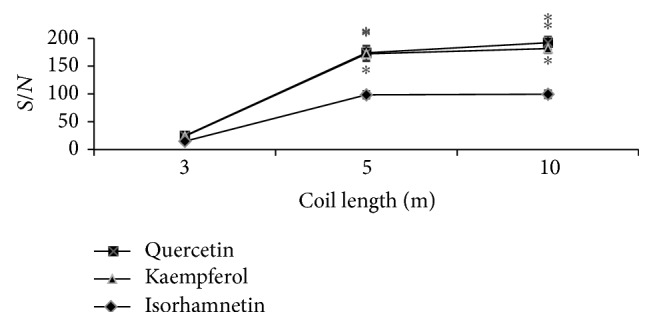
Signal-to-noise (*S*/*N*) ratios determined for quercetin, kaempferol, and isorhamnetin at different lengths of the reaction loops. *∗* indicates the significant differences at *p* < 0.05.

Statistically important (*p* < 0.05) differences were established between the *S*/*N* values of reaction loops of 3 and 5 meters' as well as 3 and 10 meters' length. However, according to the numeric values of the ratio between the peak height and baseline noise (*S*/*N*), no statistically important difference was established between reaction loops of 5 and 10 meters' length (Figure 3). The obtained results should be related to time, which is insufficient for the occurrence of a reaction between the standard compounds and the CUPRAC reagent in a 3-meter reaction loop. Meanwhile, the use of a 10-meter reaction loop in experimental investigations ensures a sufficiently long reaction time, the best *S*/*N* ratio, and minimum scattering of results.

### 3.3. Validation of HPLC Postcolumn Assays of* Ginkgo biloba* Active Antioxidant Markers

Taking into account the ICH (Q2 (R1)) [19] guidelines, several standard assay validation parameters were chosen: assay specificity, precision, minimum detectable concentration, minimum detectable amount, and linearity (Table 1). Validation was based on the area assessment of positive peaks of standard compounds in a postcolumn chromatogram.

The identification test separated the compounds by reduction activity and was used to evaluate the specificity of the HPLC-CUPRAC postcolumn method. All the chosen standard compounds were characterized by their effect on copper ion reduction. The precision of the postcolumn method was demonstrated by performing five replicate nonconsecutive injections of the four reference standard solutions on the same day (intraday) and on three different days (interday). The RSD coefficient for repeatability and intermediate precision did not exceed 1.0% (Table 1). Such precision is acceptable for all the needs of pharmaceutical analysis and is suitable for the quantitative evaluation of antioxidant markers. All 4 antioxidant compounds showed significant linear regression with a determination coefficient higher than 0.99 for the postcolumn assay. The CUPRAC postcolumn assay is fully linear over the concentration range that was tested.

### 3.4. Antioxidant Activity Assessment of* Ginkgo biloba* Medicinal Plant Raw Materials

The optimized HPLC-CUPRAC postcolumn methodology was used in further investigations of acetonic extracts of* Ginkgo biloba* leaves with the goal of evaluating the input of potential reducers of medicinal plant raw material to the antioxidant activity of raw material. It has been established that quercetin, kaempferol, and isorhamnetin, which were identified in medicinal plant raw material of* Ginkgo biloba*, possess active antioxidants (Figure 1). The antioxidant activities of the identified compounds were expressed as TEAC values and are presented in Table 2. The calculated TEAC values of bioactive compound in* Ginkgo biloba* leaves extracts confirmed that quercetin was the predominant copper reducer. Quercetin reducing activity comprises from 60 to 81% total antioxidant activity in leaf extract due to higher amounts in the raw material. Extracts of* Ginkgo biloba* leaves contain lower amounts of kaempferol and isorhamnetin; consequently these bioactive compounds determined 17–33% and 0–7% of total antioxidant activity, respectively. Activity of compounds analysed with CUPRAC postcolumn assay had the following rank order: quercetin  >  kaempferol  >  isorhamnetin. The samples collected in different districts of Lithuania were evaluated statistically. Comparison of samples of* Ginkgo biloba* leaves from different locations in Lithuania shows that the strongest reducing activity (25.99 ± 0.42) is characteristic of the raw material from the Kazlu Ruda district, whereas the lowest (4.74 ± 0.08) was from Kaunas district.

The summarized results of the reducing activity of* Ginkgo biloba* medicinal plant raw material show that quercetin, kaempferol, and isorhamnetin can be used as characteristic markers of reducing activity for quality evaluation of* Ginkgo biloba* leaves and phytopreparations.

## 4. Conclusion

The HPLC-CUPRAC postcolumn method was used in the investigation of antioxidant activity markers present in extracts of* Ginkgo biloba* medicinal raw material. This method is selective for the establishment of essential reducing compounds, such as quercetin, kaempferol, and isorhamnetin, and the control of “fingerprint” parameters.

Critical components of the HPLC-CUPRAC postcolumn methodology development are temperature of the system and flow rate of the derivatizing reagent. As the methodology of the establishment is temperature sensitive, the recommended temperature conditions for this postcolumn method should not exceed 25°C. At the eluent flow rate of 1 mL min^−1^, the recommended CUPRAC reagent flow rate was 0.2 mL min^−1^.

For future developments of similar postcolumn methodologies with CUPRAC reagent, optimization of the characteristics of the above parameters for the assessment of phytochemical composition is recommended.

## Figures and Tables

**Table 1:** Validation characteristics of the CUPRAC postcolumn assay.

Standards	Intraday RSD (%) (*n* = 5)	Interday RSD (%) (*n* = 5)	*R* ^2^	Regression equation	LOD (*µ*g mL^−1^)	LOQ (*µ*g mL^−1^)	Linearity range (*µ*g mL^−1^)
Trolox	0.04	0.08	0.999	*Y* = 2.06 · 10^7^ *x* + 9.61 · 10^4^	0.52	2.00	0.59–605
Quercetin	0.51	0.54	0.998	*Y* = 2.34 · 10^7^ *x* − 2.85 · 10^3^	0.95	3.18	4.6–74
Kaempferol	0.38	0.39	0.996	*Y* = 2.28 · 10^7^ *x* − 3.31 · 10^4^	0.66	2.22	5.2–84
Isorhamnetin	0.37	0.37	0.991	*Y* = 1.07 · 10^7^ *x* − 1.69 · 10^4^	2.37	7.92	5.3–85

RSD: relative standard deviation, *R* ^2^: correlation coefficient; LOD: limit of detection; LOQ: limit of quantitation.

**Table 2:** TEAC values (*µ*mol g^−1^ of dry weight) of *Ginkgo biloba* L. leaf in CUPRAC postcolumn assay.

Peak number	Analytes	Cultivation source							
		*T*_*R*_ (min)	Sample 1	Sample 2	Sample 3	Sample 4	Sample 5	Sample 6	Sample 7
1	Quercetin	13.7	6.79 ± 0.12^b^	11.19 ± 0.2^c^	3.04 ± 0.04^a^	12.22 ± 0.18^d^	19.50 ± 0.1^f^	3.12 ± 0.04^a^	13.74 ± 0.18^e^
2	Kaempferol	19.2	2.48 ± 0.05^b^	2.40 ± 0.1^b^	1.44 ± 0.03^a^	3.12 ± 0.04^c^	5.59 ± 0.3^e^	1.68 ± 0.01^a^	3.98 ± 0.05^d^
3	Isorhamnetin	20.4	nd	0.37 ± 0.01^bc^	0.26 ± 0.01^a^	0.40 ± 0.01^cd^	0.90 ± 0.03^e^	0.44 ± 0.02^d^	0.32 ± 0.01^b^
Total			9.27 ± 0.16^b^	13.96 ± 0.30^c^	4.74 ± 0.08^a^	15.74 ± 0.25^d^	25.99 ± 0.42^f^	5.24 ± 0.07^a^	18.04 ± 0.27^e^

*T*_*R*_: retention time; nd: not detected.

*Notes.* (1) Averages marked in different letters in the lines show statistically significant difference (at *p* < 0.05).

(2) Values are expressed as mean quantities with standard deviation.
